# Supplementary material for: Pyruvate Dehydrogenase Kinase 1 inhibition mediated oxidative phosphorylation enhancement in cartilage promotes osteoarthritis progression
Source: BMC Musculoskelet Disord. 2023 Jul 20;24:597. doi: 10.1186/s12891-023-06585-6 (PMC10357736; doi:10.1186/s12891-023-06585-6)
Supplement: Supplementary file 1 — Additional file 1. [file 12891_2023_6585_MOESM1_ESM.zip › BP.Gsea.1653623667859/GOBP_PROTEIN_LOCALIZATION_TO_ENDOPLASMIC_RETICULUM.html]

Details for gene set GOBP\_PROTEIN\_LOCALIZATION\_TO\_ENDOPLASMIC\_RETICULUM[GSEA]

|  || Dataset | 55457\_collapsed\_to\_symbols.cls.cls #OA\_versus\_normal.cls.cls #OA\_versus\_normal\_repos |
| Phenotype | cls.cls#OA\_versus\_normal\_repos |
| Upregulated in class | OA |
| GeneSet | GOBP\_PROTEIN\_LOCALIZATION\_TO\_ENDOPLASMIC\_RETICULUM |
| Enrichment Score (ES) | 0.43552563 |
| Normalized Enrichment Score (NES) | 1.7860076 |
| Nominal p-value | 0.0 |
| FDR q-value | 0.08934988 |
| FWER p-Value | 0.989 |
Table: GSEA Results Summary

  

Fig 1: Enrichment plot: GOBP\_PROTEIN\_LOCALIZATION\_TO\_ENDOPLASMIC\_RETICULUM      
 Profile of the Running ES Score & Positions of GeneSet Members on the Rank Ordered List

  

| SYMBOL | TITLE | RANK IN GENE LIST | RANK METRIC SCORE | RUNNING ES | CORE ENRICHMENT || 1 | GPAA1 | glycosylphosphatidylinositol anchor attachment 1 [Source:HGNC Symbol;Acc:HGNC:4446] | 44 | 1.006 | 0.0525 | Yes |
| 2 | KDELR3 | KDEL endoplasmic reticulum protein retention receptor 3 [Source:HGNC Symbol;Acc:HGNC:6306] | 77 | 0.950 | 0.1028 | Yes |
| 3 | INSIG2 | insulin induced gene 2 [Source:HGNC Symbol;Acc:HGNC:20452] | 205 | 0.814 | 0.1382 | Yes |
| 4 | SPCS1 | signal peptidase complex subunit 1 [Source:HGNC Symbol;Acc:HGNC:23401] | 303 | 0.747 | 0.1721 | Yes |
| 5 | UBL4A | ubiquitin like 4A [Source:HGNC Symbol;Acc:HGNC:12505] | 464 | 0.675 | 0.1972 | Yes |
| 6 | CHMP4A | charged multivesicular body protein 4A [Source:HGNC Symbol;Acc:HGNC:20274] | 526 | 0.649 | 0.2285 | Yes |
| 7 | TRAM1 | translocation associated membrane protein 1 [Source:HGNC Symbol;Acc:HGNC:20568] | 569 | 0.633 | 0.2604 | Yes |
| 8 | RER1 | retention in endoplasmic reticulum sorting receptor 1 [Source:HGNC Symbol;Acc:HGNC:30309] | 724 | 0.588 | 0.2811 | Yes |
| 9 | GABARAPL2 | GABA type A receptor associated protein like 2 [Source:HGNC Symbol;Acc:HGNC:13291] | 776 | 0.575 | 0.3091 | Yes |
| 10 | RYR2 | ryanodine receptor 2 [Source:HGNC Symbol;Acc:HGNC:10484] | 828 | 0.561 | 0.3363 | Yes |
| 11 | GET3 | "guided entry of tail-anchored proteins factor 3, ATPase [Source:HGNC Symbol;Acc:HGNC:752]" | 1161 | 0.485 | 0.3374 | Yes |
| 12 | SEC61G | SEC61 translocon subunit gamma [Source:HGNC Symbol;Acc:HGNC:18277] | 1163 | 0.485 | 0.3642 | Yes |
| 13 | SEC62 | "SEC62 homolog, preprotein translocation factor [Source:HGNC Symbol;Acc:HGNC:11846]" | 1272 | 0.464 | 0.3816 | Yes |
| 14 | KDELR2 | KDEL endoplasmic reticulum protein retention receptor 2 [Source:HGNC Symbol;Acc:HGNC:6305] | 1486 | 0.431 | 0.3890 | Yes |
| 15 | BCAP29 | B cell receptor associated protein 29 [Source:HGNC Symbol;Acc:HGNC:24131] | 1548 | 0.421 | 0.4076 | Yes |
| 16 | BCAP31 | B cell receptor associated protein 31 [Source:HGNC Symbol;Acc:HGNC:16695] | 1584 | 0.416 | 0.4280 | Yes |
| 17 | RAB3GAP1 | RAB3 GTPase activating protein catalytic subunit 1 [Source:HGNC Symbol;Acc:HGNC:17063] | 1766 | 0.389 | 0.4355 | Yes |
| 18 | KDELR1 | KDEL endoplasmic reticulum protein retention receptor 1 [Source:HGNC Symbol;Acc:HGNC:6304] | 2227 | 0.336 | 0.4184 | No |
| 19 | SPCS3 | signal peptidase complex subunit 3 [Source:HGNC Symbol;Acc:HGNC:26212] | 2772 | 0.273 | 0.3912 | No |
| 20 | MIA3 | MIA SH3 domain ER export factor 3 [Source:HGNC Symbol;Acc:HGNC:24008] | 3550 | 0.198 | 0.3417 | No |
| 21 | TRAM2 | translocation associated membrane protein 2 [Source:HGNC Symbol;Acc:HGNC:16855] | 4913 | 0.079 | 0.2401 | No |
| 22 | BAG6 | BAG cochaperone 6 [Source:HGNC Symbol;Acc:HGNC:13919] | 5023 | 0.071 | 0.2356 | No |
| 23 | ANK2 | ankyrin 2 [Source:HGNC Symbol;Acc:HGNC:493] | 5320 | 0.046 | 0.2151 | No |
| 24 | SEC61A1 | SEC61 translocon subunit alpha 1 [Source:HGNC Symbol;Acc:HGNC:18276] | 5378 | 0.041 | 0.2130 | No |
| 25 | OS9 | OS9 endoplasmic reticulum lectin [Source:HGNC Symbol;Acc:HGNC:16994] | 5418 | 0.039 | 0.2121 | No |
| 26 | SRP9 | signal recognition particle 9 [Source:HGNC Symbol;Acc:HGNC:11304] | 5498 | 0.033 | 0.2078 | No |
| 27 | EDEM1 | ER degradation enhancing alpha-mannosidase like protein 1 [Source:HGNC Symbol;Acc:HGNC:18967] | 5633 | 0.021 | 0.1985 | No |
| 28 | SSR3 | signal sequence receptor subunit 3 [Source:HGNC Symbol;Acc:HGNC:11325] | 5969 | -0.002 | 0.1726 | No |
| 29 | BTF3 | basic transcription factor 3 [Source:HGNC Symbol;Acc:HGNC:1125] | 6033 | -0.007 | 0.1681 | No |
| 30 | HERPUD1 | homocysteine inducible ER protein with ubiquitin like domain 1 [Source:HGNC Symbol;Acc:HGNC:13744] | 6520 | -0.044 | 0.1326 | No |
| 31 | SRPRA | SRP receptor subunit alpha [Source:HGNC Symbol;Acc:HGNC:11307] | 6566 | -0.047 | 0.1318 | No |
| 32 | SRP19 | signal recognition particle 19 [Source:HGNC Symbol;Acc:HGNC:11300] | 6945 | -0.075 | 0.1065 | No |
| 33 | SPCS2 | signal peptidase complex subunit 2 [Source:HGNC Symbol;Acc:HGNC:28962] | 6981 | -0.078 | 0.1081 | No |
| 34 | TAPT1 | transmembrane anterior posterior transformation 1 [Source:HGNC Symbol;Acc:HGNC:26887] | 7256 | -0.100 | 0.0924 | No |
| 35 | SRPRB | SRP receptor subunit beta [Source:HGNC Symbol;Acc:HGNC:24085] | 7553 | -0.123 | 0.0762 | No |
| 36 | GRIK5 | glutamate ionotropic receptor kainate type subunit 5 [Source:HGNC Symbol;Acc:HGNC:4583] | 7912 | -0.148 | 0.0566 | No |
| 37 | RAB3GAP2 | RAB3 GTPase activating non-catalytic protein subunit 2 [Source:HGNC Symbol;Acc:HGNC:17168] | 7957 | -0.152 | 0.0616 | No |
| 38 | SEC63 | "SEC63 homolog, protein translocation regulator [Source:HGNC Symbol;Acc:HGNC:21082]" | 8151 | -0.167 | 0.0558 | No |
| 39 | SRP72 | signal recognition particle 72 [Source:HGNC Symbol;Acc:HGNC:11303] | 8338 | -0.181 | 0.0514 | No |
| 40 | GBF1 | golgi brefeldin A resistant guanine nucleotide exchange factor 1 [Source:HGNC Symbol;Acc:HGNC:4181] | 8461 | -0.191 | 0.0525 | No |
| 41 | SEC61B | SEC61 translocon subunit beta [Source:HGNC Symbol;Acc:HGNC:16993] | 8510 | -0.194 | 0.0596 | No |
| 42 | VAPA | VAMP associated protein A [Source:HGNC Symbol;Acc:HGNC:12648] | 8854 | -0.221 | 0.0452 | No |
| 43 | PDIA2 | protein disulfide isomerase family A member 2 [Source:HGNC Symbol;Acc:HGNC:14180] | 8961 | -0.229 | 0.0496 | No |
| 44 | SRP14 | signal recognition particle 14 [Source:HGNC Symbol;Acc:HGNC:11299] | 9288 | -0.255 | 0.0384 | No |
| 45 | SEC16A | "SEC16 homolog A, endoplasmic reticulum export factor [Source:HGNC Symbol;Acc:HGNC:29006]" | 9434 | -0.268 | 0.0420 | No |
| 46 | DDRGK1 | DDRGK domain containing 1 [Source:HGNC Symbol;Acc:HGNC:16110] | 9741 | -0.296 | 0.0346 | No |
| 47 | MAN1A1 | mannosidase alpha class 1A member 1 [Source:HGNC Symbol;Acc:HGNC:6821] | 10207 | -0.341 | 0.0174 | No |
| 48 | INSIG1 | insulin induced gene 1 [Source:HGNC Symbol;Acc:HGNC:6083] | 10264 | -0.347 | 0.0323 | No |
| 49 | HSPA5 | heat shock protein family A (Hsp70) member 5 [Source:HGNC Symbol;Acc:HGNC:5238] | 10820 | -0.407 | 0.0118 | No |
| 50 | SGTA | small glutamine rich tetratricopeptide repeat co-chaperone alpha [Source:HGNC Symbol;Acc:HGNC:10819] | 11185 | -0.455 | 0.0088 | No |
| 51 | SEC61A2 | SEC61 translocon subunit alpha 2 [Source:HGNC Symbol;Acc:HGNC:17702] | 11352 | -0.479 | 0.0225 | No |
| 52 | SRP54 | signal recognition particle 54 [Source:HGNC Symbol;Acc:HGNC:11301] | 11754 | -0.541 | 0.0213 | No |
| 53 | RTN4 | reticulon 4 [Source:HGNC Symbol;Acc:HGNC:14085] | 12017 | -0.596 | 0.0341 | No |
| 54 | PPP1R15A | protein phosphatase 1 regulatory subunit 15A [Source:HGNC Symbol;Acc:HGNC:14375] | 12107 | -0.622 | 0.0617 | No |
Table: GSEA details [plain text format]

  

Fig 2: GOBP\_PROTEIN\_LOCALIZATION\_TO\_ENDOPLASMIC\_RETICULUM      
 Blue-Pink O' Gram in the Space of the Analyzed GeneSet

  

Fig 3: GOBP\_PROTEIN\_LOCALIZATION\_TO\_ENDOPLASMIC\_RETICULUM: Random ES distribution      
 Gene set null distribution of ES for **GOBP\_PROTEIN\_LOCALIZATION\_TO\_ENDOPLASMIC\_RETICULUM**
